# Polyphosphate-based strategies in enamel remineralization: mechanisms, evidence, and clinical potential

**DOI:** 10.3389/froh.2026.1801636

**Published:** 2026-04-20

**Authors:** Btissam Seffahi, Imane El Ouarti, Faïza Abdallaoui

**Affiliations:** Department of Conservative Dentistry and Endodontics, Faculty of Dental Medicine, Mohammed V University, Rabat, Morocco

**Keywords:** calcium glycerophosphate, dental caries, enamel remineralization, polyphosphates, sodium hexametaphosphate, sodium trimetaphosphate

## Abstract

Minimally invasive management of initial enamel caries focuses on strategies that promote subsurface remineralization while limiting fluoride exposure, particularly in children. Phosphate salts such as sodium trimetaphosphate (TMP), sodium hexametaphosphate (SHMP) and calcium glycerophosphate (CaGP) have therefore been incorporated into topical fluoride formulations as interfacial modifiers. This narrative review critically examines mechanisms, experimental evidence and clinical data on polyphosphate systems for early enamel carious lesions. Relevant literature from databases including PubMed, Scopus, Cochrane and ScienceDirect was reviewed, focusing on *in vitro*, *in situ* and *in vivo* human studies including clinical trials evaluating TMP-, SHMP- or CaGP-containing products. Available evidence indicates that these salts adsorb to enamel, assemble ion-rich surface layers, modulate biofilm acidogenicity and favor deeper subsurface repair, with nano-sized forms amplifying these effects. Across toothpastes, gels and varnishes, *in vitro* and *in situ* studies suggest that several low-fluoride polyphosphate formulations may achieve mineral uptake comparable to conventional 1,100-ppm F products, when fluoride:polyphosphate ratios were kept within a favorable window. Available clinical trials, although few and heterogeneous, suggest caries reduction in children using low-fluoride toothpastes containing polyphosphate salts. Overall, polyphosphate systems may represent a relevant option for subsurface enamel remineralization in susceptible groups, while longer-term clinical evaluation is still needed.

## 1 Introduction

Dental caries remain one of the most prevalent chronic diseases worldwide, affecting all ages and imposing a significant public health burden (1). The disease reflects a dynamic balance between demineralization and remineralization at the tooth surface, driven largely by acidogenic biofilms in the presence of fermentable carbohydrates (2). Caries usually begin as subsurface lesions beneath the enamel surface, where organic acids produced by dental biofilm bacteria from dietary sugars accumulate in the plaque fluid and lower the pH at the biofilm-enamel interface (3). When the pH falls below a critical level, the fluid becomes undersaturated and enamel mineral starts to dissolve. The surface layer partially demineralizes and becomes more porous, allowing acids and dissolved ions to diffuse inward so that mineral loss is concentrated in a subsurface zone. As this process continues without effective control of the causative factors, the subsurface lesion enlarges and, once sufficient mineral has been lost, becomes visible clinically as a white-spot lesion and can eventually progress to cavitation (3, 4).

Owing to the dynamic nature of the disease process, these very early subclinical lesions can still be arrested or reversed, especially in the presence of fluoride (3). Once sugars are cleared from the mouth by swallowing and salivary dilution, plaque acids are buffered by saliva, the pH of the biofilm fluid rises towards neutrality and the fluid becomes more saturated with calcium, phosphate and fluoride ions. Under these conditions, demineralization stops and mineral is redeposited, with remineralization mainly affecting the outer enamel surface, while repair of the deeper subsurface lesion remains limited (3–5).

For decades, topical fluoride has played a central role in caries prevention, by inhibiting demineralization, enhancing remineralization and affecting the metabolism of cariogenic bacteria (6). Delivered in familiar forms such as toothpastes, gels and mouthrinses, it has contributed to a substantial decrease in caries prevalence (7). At the crystal level, fluoride can be incorporated into the enamel hydroxyapatite and form calcium fluoride (CaF₂)-like surface deposits that release fluoride during subsequent acid challenges, thereby strengthening and rehardening the enamel surface (8). However, these effects are largely confined to the outer layer and may not fully repair established subsurface lesions, especially under a persistent cariogenic challenge (3, 9). In addition, concerns about fluoride overexposure have been raised. Dental fluorosis, a developmental enamel defect resulting from excessive fluoride intake during enamel formation, represents a well-recognized consequence of such overexposure (10). Possible neurodevelopmental effects associated with high systemic fluoride exposure have also been reported (11), although this issue remains debated and fluoride is widely considered safe and effective for caries prevention when used at recommended levels (12, 13). These concerns have driven the search for safer, complementary remineralization strategies, especially for high-risk patients such as children and individuals with reduced salivary flow.

To address these limitations, researchers have explored combining fluoride with bioavailable sources of calcium and phosphate (e.g., CPP-ACP) to enhance remineralization (14). More recently, polyphosphate-based systems, notably sodium trimetaphosphate (TMP), sodium hexametaphosphate (SHMP), and calcium glycerophosphate (CaGP) have received growing attention in enamel research, with studies investigating both micrometric and nanometric formulations (15–17). Although polyphosphates themselves are not new, their applications in remineralization, particularly in *in vitro* and *in situ* models, are being actively explored for their ability to interact with the enamel surface, modulate mineral dynamics, and potentially enhance fluoride efficacy (9, 18, 19). Within this context, the present review examines polyphosphate-based systems as adjunctive fluoride-enhancing systems, summarizing current evidence on their mechanisms of action, *in vitro* and *in situ* outcomes, and potential relevance to modern remineralization strategies.

## 2 Methods

### 2.1 Literature search

This work was conducted as a narrative review aimed at summarizing the available evidence on polyphosphate-based strategies for enamel remineralization. The literature search was conducted in PubMed, Scopus, the Cochrane Library, and ScienceDirect using combinations of keywords such as (“trimetaphosphate” OR “TMP” OR “sodium hexametaphosphate” OR “SHMP” OR “calcium glycerophosphate” OR “CaGP” OR polyphosphate) AND (“enamel remineralization” OR “dental caries”). No restrictions were applied regarding publication year or language, and the final search was run on 30 November 2025. Titles and abstracts were screened by two reviewers, followed by full-text assessment based on predefined inclusion criteria. Reference lists of included articles were also screened to identify additional relevant studies.

### 2.2 Eligibility criteria

Studies investigating polyphosphate salts, including TMP, SHMP, and CaGP, in the remineralization of early enamel carious lesions were considered. *In vitro* studies, *in situ* studies, as well as *in vivo* human studies, were included when they evaluated these compounds alone or in combination with fluoride or other remineralizing agents. Studies focusing on dentine remineralization, enamel erosion, bleaching applications, or developmental enamel defects such as fluorosis, molar–incisor hypomineralization, or amelogenesis imperfecta were excluded. Case reports, case series, commentaries, cross-sectional studies, and letters to the editor were also excluded.

### 2.3 Study selection

Titles and abstracts were reviewed for relevance, followed by examination of the full texts of potentially relevant articles. The selection process was conducted independently by two reviewers, and disagreements were resolved through discussion. A total of 66 studies met the inclusion criteria, including 35 *in vitro* studies, 20 *in situ* studies, and 11 *in vivo* human studies.

### 2.4 Data extraction and synthesis

Relevant information from the included studies, such as study design, investigated compounds, models, and reported remineralization outcomes, was reviewed. The evidence was then synthesized narratively.

As this work represents a narrative review based on a focused but non-systematic literature search and without a predefined protocol or formal risk-of-bias assessment, the findings should be interpreted with consideration of potential methodological heterogeneity and selection bias.

## 3 Enamel remineralization: rationale and limits

Dental enamel stands out as the most highly mineralized tissue in the human body, consisting of about 96% mineral by weight, predominantly in the form of carbonated hydroxyapatite crystals (5, 20). After eruption, enamel is acellular and lacks biological repair mechanisms, which makes it particularly vulnerable to sustained acidic challenges in the oral environment (3, 21). Nevertheless, post-eruptive mineral gain occurs through physicochemical remineralization processes governed by pH-driven cycles at the enamel surface: below the critical pH (∼5.5) hydroxyapatite dissolves, when pH returns towards neutrality and the fluid is supersaturated with calcium and phosphate ions, mineral can redeposit principally via saliva, with topical fluoride enhancing this process (3).

Initial enamel caries present as a subsurface demineralized lesion beneath an intact, highly mineralized surface layer, which restricts ion diffusion into the lesion body (3). Although saliva supplies calcium and phosphate ions that contribute to surface remineralization, this process may be insufficient to halt lesion progression under conditions such as frequent sugar intake, persistent biofilm or reduced salivary flow (21). On the other hand, fluoride improves acid resistance in two main ways: at low, sustained concentrations in plaque and saliva it becomes incorporated into enamel hydroxyapatite (fluoride-substituted apatite), whereas brief high-concentration peaks after topical application generate calcium fluoride-like (CaF₂) surface deposits that act as fluoride source during subsequent acid challenges (22). These effects, however, are typically strongest at or near the enamel surface, and several investigations describe fluoride's remineralizing action as primarily superficial with limited reach into deeper subsurface pores (8). In addition, forming and stabilizing fluoride-substituted apatite require adequate local supplies of calcium ions (Ca^2^^+^) and phosphate ions (PO₄^3^^−^). As a consequence, ion availability may not reach levels required for effective remineralization under everyday conditions, particularly with hyposalivation (9, 21). These limitations underscore a therapeutic gap that motivates adjunct approaches to position and stabilize calcium, phosphate and fluoride at the enamel interface and promote their controlled inward diffusion into the demineralized subsurface lesion.

## 4 Overview of polyphosphate systems

Polyphosphates are condensed phosphate compounds composed of orthophosphate (PO₄^3^^−^) units linked through phosphoanhydride (P–O–P) bonds, forming either linear chains or cyclic structures. In oral care research, several polyphosphate and phosphate-based systems have been investigated as adjuncts to fluoride, including TMP, SHMP, and CaGP. These compounds can interact with enamel surfaces and may influence demineralization–remineralization processes (8, 23).

### 4.1 Sodium trimetaphosphate (TMP)

TMP (Na₃P₃O₉) is a cyclic inorganic condensed phosphate (18). Among polyphosphates studied in oral care, TMP has shown the most consistent anticaries effects in the available literature, not only inhibiting demineralization but also enhancing remineralization (24, 25). It additionally exhibits high-affinity, long-lasting adsorption to phosphate sites on enamel, persisting longer than other polyphosphates, therefore prolonging its interfacial effect (26–30).

### 4.2 Sodium hexametaphosphate (SHMP)

SHMP (Na₆P₆O₁₈) is a cyclic inorganic metaphosphate. The term “SHMP” however, is also used for glassy, predominantly linear sodium polyphosphates. Even though modern nomenclature reserves “meta” for cyclic compounds, this has created recurring confusion in the literature. In this review, SHMP refers to the cyclic compound (18, 31, 32). In oral care, it has been incorporated into toothpastes as an anti-tartar/whitening agent through adsorption to enamel and has shown anti-biofilm activity; alongside fluoride, *in vitro* and *in situ* studies on carious enamel lesions report reduced demineralization, supporting an adjunct role in caries prevention. Historically, SHMP has been employed in foods as an antimicrobial by increasing bacterial outer-membrane permeability (33, 34).

### 4.3 Calcium glycerophosphate (CaGP)

CaGP (C₃H₇CaO₆P) is an organic calcium phosphate salt with established anticariogenic properties. It occurs in three isomeric forms (*α*, *β*, *γ*), with *β*-CaGP exhibiting greater release of calcium and phosphate ions (35, 36). Its anticaries potential was first shown when used as a food additive, demonstrating protection in animal and human studies, and was followed by early clinical trials in toothpaste formulations (37–40). CaGP has since drawn interest for supplying bioavailable calcium and phosphate ions, contributing to pH modulation and influencing bacterial metabolism (41), which has led to its incorporation into products such as fluoride toothpaste and glass-ionomer cements where it continues to be investigated across *in vitro*, *in situ* and clinical studies (17, 19, 36, 42–48).

## 5 Mechanisms of action

Current mechanistic understanding of polyphosphate-based systems in enamel remineralization derives predominantly from *in vitro* and *in situ* studies. Polyphosphate-based systems have been suggested to contribute to enamel remineralization through combined physico-chemical interactions with hydroxyapatite and modulation of local ionic and microbial conditions. These salts are thought to share several common mechanisms, as reported in *in vitro* and *in situ* studies: they adsorb to the enamel surface through multiple phosphate binding sites, reduce hydroxyapatite solubility, and assemble interfacial layers that may function as selectively permeable barriers to acid diffusion while facilitating inward movement of remineralizing ions (23, 31, 42). By retaining Ca^2^^+^ and calcium monofluoride ion (CaF^+^) at the interface and releasing them in a controlled way during pH drops, polyphosphates may prevent immediate surface precipitation (e.g., CaF₂-like deposits), maintain permeability, and promote neutral species such as neutral calcium hydrogenphosphate (CaHPO₄⁰) and undissociated hydrogen fluoride (HF⁰) that are thought to diffuse more efficiently into subsurface lesions than charged ions, potentially shifting remineralization from an early surface-confined response to a later, deeper repair within the lesion body (19, 27, 44, 49). In the biofilm, polyphosphates may bind metal ions and may contribute to plaque buffering through phosphate release. Owing to their high affinity for hydroxyapatite, they may also indirectly interfere with microbial metabolism by limiting adsorption of organic material and bacteria at the tooth surface, which may lower extracellular polysaccharide synthesis (EPS). In SHMP and CaGP-based formulations, these surface effects have been associated in *in vitro* and *in situ* studies with reduced *Streptococcus mutans* counts and diminished glycolytic activity, which may contribute to lower biofilm acidogenicity. Collectively, these effects may shift the de-/re-mineralization balance toward subsurface repair and may enhance fluoride's remineralizing effect, with *in vitro* and *in situ* studies reporting comparable or improved outcomes at lower fluoride exposure (50–57).

### 5.1 Mechanism of action of TMP

TMP is thought to adsorb to enamel via its negatively charged phosphate groups at phosphate sites on the enamel surface, assembling a thin interfacial film (protective layer) that may decrease calcium and phosphate precipitation at the enamel pores and restrict acid diffusion (54). This film may act as a selectively permeable interface: while it may limit acid penetration, it may still allow inward diffusion of Ca^2^^+^ and F^−^, supporting ion delivery into the lesion body and a more pronounced subsurface effect (23, 51). The behavior has been attributed to TMP's ability to remain bound to enamel for a longer period than other polyphosphates, which may retain Ca^2^^+^/CaF^+^ at the interface and may enable controlled release of neutral, more-diffusible species during pH drops (18, 23, 52, 55, 57). At the pellicle-biofilm interface, TMP may alter the affinity between enamel and salivary proteins, leading to less EPS accumulation, thereby contributing to protection against demineralization (53, 56). Because TMP appears to act primarily by conditioning the enamel surface, pairing it with CPP-ACPF may provide the ion supply and stabilization needed. The combination has been reported to produce synergistic effects, enhanced nucleation, a thicker, more stable reticular layer, and improved subsurface remineralization at lower fluoride levels (26, 50, 53, 54).

### 5.2 Mechanism of action of SHMP

SHMP is a negatively charged polyphosphate that does not undergo spontaneous hydrolysis in the oral environment and is therefore considered not to be a direct phosphate donor to enamel (58). Like TMP, it is thought to exhibit high affinity for hydroxyapatite via multiple binding sites. Once adsorbed, it may form stable complexes with calcium (Ca^2^^+^), magnesium (Mg^2^^+^), potassium (K^+^), aluminum (Al^3^^+^), and ferric iron (Fe^3^^+^) ions, with sodium ions (Na^+^) in the ring readily replaced, thereby contributing to the same reticular interfacial layer described above. Under cariogenic challenge, this layer may behave as previously outlined: it may limit acid diffusion without impeding inward diffusion of remineralizing ions, sustaining the depth-focused profile. In parallel, SHMP may influence biofilm metabolism by the same route described for TMP, which has been linked to reduced biofilm acidogenicity and cariostatic effects (31, 59–62).

### 5.3 Mechanism of action of CaGP

CaGP differs from the other polyphosphates in being an organic calcium phosphate salt that can directly supply bioavailable calcium and phosphate for remineralization (42, 43). Its molecular structure provides two binding sites for interaction with hydroxyapatite, which may favor adsorption to the enamel surface (17). Once adsorbed, its phosphate groups may be neutralized by Ca^2^^+^ and hydroxyl ions (OH^−^) drawn from the lattice, and the Ca–P association in CaGP, being the least stable part of the molecule, may be disrupted, promoting calcium release to the medium and leaving the anionic phosphate group bound at the interface (17, 36, 44). This calcium release increases the ionic activity of neutral species, which may enhance subsurface remineralization. In the biofilm, CaGP may undergo enzymatic hydrolysis by bacterial phosphatases, generating phosphate species such as hydrogen phosphate ion (HPO₄^2^^−^) that may help buffer plaque pH, while also interfering with bacterial metabolism and reducing extracellular polysaccharides and acidogenicity (47, 63, 64).

### 5.4 Mechanism of action of nano-polyphosphates

The mechanisms described above may be strengthened when polyphosphates are used in nano-sized form (nano-TMP, nano-SHMP, nano-CaGP). Owing to a much higher surface-area-to-volume ratio and a greater fraction of surface atoms, nanoparticles may be more reactive than micrometric particles and may adsorb more efficiently to enamel (15, 17, 27). Once at the interface, nano-polyphosphates may retain greater amounts of Ca^2^^+^ and CaF^+^ within their negatively charged structure, which may stabilize the interfacial layer, reduce premature surface precipitation (e.g., CaF₂-like deposits) and pore obstruction, and thereby favor inward ion diffusion with a more pronounced subsurface effect (36, 55, 65). These nanoscale thermodynamic/kinetic advantages may translate into higher stability and efficiency of the interfacial film and may permit comparable remineralizing action at lower polyphosphate concentrations (58, 66–71).

## 6 Discussion

Evidence from *in vitro* and *in situ* studies suggests that combining fluoride with polyphosphates at reduced fluoride levels may lower enamel demineralization and promote remineralization, particularly within the deeper zones of initial enamel lesions (72). Most evidence comes from toothpaste formulations; adding TMP, SHMP or CaGP to fluoride has been associated with improved surface and subsurface hardness and increased calcium, phosphate and fluoride uptake compared with fluoride alone under the same conditions. Across these *in vitro* and *in situ* toothpaste studies, low-fluoride formulations containing TMP, SHMP or CaGP often showed favorable hardness and mineral uptake outcomes relative to a 1,100-ppm F toothpaste, and in some cases (e.g., 3% TMP with 1,100 ppm F) higher hardness and mineral uptake values were reported compared with the 1,100-ppm F control (16, 31, 42, 43, 45, 60, 64, 72–74). Alongside their impact on enamel, these formulations have also been evaluated for their effects on the biofilm. In these studies, biofilms formed in the presence of polyphosphate-fluoride toothpastes retained more Ca^2+^ and F^−^ during cariogenic challenges, formed less insoluble EPS and maintained higher pH after sucrose exposure than controls, indicating a less acidogenic environment at the tooth–biofilm interface (61, 63, 64, 66, 67). In addition to these *in vitro* and *in situ* findings, limited clinical evidence is available. An 18-month randomized clinical trial in children reported lower caries increment with a 500-ppm F toothpaste supplemented with TMP and CaGP than with a conventional 1,100-ppm F toothpaste (19). Another noteworthy finding is that TMP-containing fluoride toothpastes did not enhance remineralization in the outer enamel layers and were associated with reduced precipitation of CaF₂ and firmly bound fluoride at the surface. This has been interpreted as potentially favoring the diffusion of neutral species into enamel and thereby promoting repair within the subsurface lesion (24, 27, 51, 57, 67). This is particularly relevant because this region may lose up to 50% of its original mineral content while remaining covered by an apparently intact surface layer (5), suggesting that phosphate salts may be of interest as adjuvants in low-fluoride toothpaste formulations (75).

Similar patterns have been reported when polyphosphates are delivered in other vehicles, including gels, varnishes and mouthrinses (57, 76, 77). In *in vitro* and *in situ* studies using gels, combining 4,500-ppm F with higher concentrations of TMP or SHMP significantly reduced mineral loss, with effects comparable to highly concentrated acidulated phosphate fluoride gels (12,300-ppm F) (66, 68, 69). For varnishes, *in vitro* and *in situ* studies have likewise reported enhanced remineralization when 5% TMP was incorporated into fluoride formulations compared with fluoride alone (57, 78).

Another factor that appears to modulate these effects is particle size. As polyphosphates act primarily by adsorbing to enamel and retaining calcium and fluoride at the enamel surface, reducing particle size may be more effective than increasing phosphate concentration (15). Numerous *in vitro* and *in situ* studies have shown that nano-TMP, nano-SHMP and nano-CaGP achieved greater remineralization, less mineral loss and higher calcium and phosphate uptake than micrometric salts or fluoride alone at the same fluoride concentration (15, 66, 69, 79), and they more efficiently lowered EPS and acidogenicity in biofilm models, suggesting improved performance relative to their conventional counterparts (17, 27, 58, 62, 67, 80). This effect has been attributed to their higher reactivity and retention of Ca^2^^+^ and CaF^+^ within the interfacial layer, reducing fluoride precipitation at the outer surface and favoring diffusion of ions into the lesion body and a more extensive subsurface repair (36, 55, 65). In this context, nano-polyphosphate formulations may represent a potential approach, since their properties could allow reductions in phosphate salt concentration and fluoride levels while maintaining remineralizing effects (15, 17).

All these benefits, however, appear to depend on maintaining the fluoride:polyphosphate molar ratio within a favorable window. Because polyphosphates adsorb rapidly at the enamel surface, experimental work has shown that excess anionic phosphate can outcompete fluoride and sequester Ca^2^^+^/CaF^+^, reducing fluoride binding and ion transport into the lesion (17, 60, 72). For TMP, the most favorable outcomes have been reported around a TMP/F ratio of ≈1.7 (3% TMP with 1,100 ppm F), whereas higher ratios (≈2.5–5.1) have been associated with reduced TMP–F synergy and lower enamel fluoride levels (72–74). For SHMP, a similar SHMP/F window has been identified, with proportions corresponding to 1% SHMP with 1,100 ppm F showing the most favorable outcomes, while higher SHMP/F ratios (e.g., ≥2% SHMP at the same F level) have been linked to increased demineralization (31, 60, 72). When it comes to CaGP, studies in low-fluoride toothpastes indicate that a F:CaGP ratio of about 2.2:1 (0.25% CaGP with 500 ppm F) provides the most favorable outcomes, whereas off-ratio combinations reduce enamel fluoride and offer no additional gain (36, 43, 48). Consequently, fluoride–polyphosphate systems should operate within a defined molar ratio to maintain fluoride availability and effective mineral incorporation.

To optimize these systems further, several studies have combined polyphosphates, mainly TMP, with additional calcium and phosphate sources. *In vitro* and *in situ* models have shown that formulations containing fluoride in combination with TMP and CPP-ACP generally demonstrate greater hardness recovery and mineral uptake than fluoride and TMP or fluoride and CPP-ACP alone (26, 50, 53, 54), and that low-fluoride toothpastes with TMP plus calcium citrate or TMP with xylitol/erythritol have shown comparable outcomes to those observed with 1,100-ppm F controls (81–83). These findings may be explained by a model in which fluoride stabilizes apatite, phosphate salts organize the interfacial layer and favor inward ion transport, and additional components supply and stabilize calcium and phosphate, together enhancing subsurface repair at lower fluoride levels (26). However, the same requirement for an appropriate fluoride:polyphosphate molar ratio applies to these combinations.

Although *in vitro*, *in situ* and limited *in vivo* human data suggest potential benefit, results are not fully consistent across studies. Some experiments did not confirm an advantage of polyphosphate-containing formulations over highly concentrated fluoride gels, conventional fluoride toothpastes, or placebo, and in some cases no change in biofilm-fluid fluoride or calcium was observed despite earlier positive enamel outcomes (68, 84). Clinically, low-fluoride toothpastes with TMP or CaGP reduced caries in some trials (40, 85–89), whereas others found no additional benefit when polyphosphates were added (90–92). Most clinical trials to date have been conducted in children with increased caries risk and have focused on non-cavitated enamel lesions, so direct extrapolation to adult populations and to other high-risk conditions such as xerostomia or root caries should be made with caution. This heterogeneity highlights the need for further clinical trials to determine under which conditions polyphosphate systems may support the management or reversal of non-cavitated lesions and limit further demineralization. Despite these uncertainties, polyphosphate systems have been incorporated into some commercially available oral care products. SHMP appears to be the polyphosphate most widely used, mainly in anti-stain and anti-calculus formulations, whereas formulations that use phosphate salts primarily for plaque buffering and remineralization enhancement appear less common and are supported by limited publicly available information on their composition and long-term clinical performance (9).

Overall, current evidence suggests that polyphosphate-fluoride formulations may enhance the action of calcium, phosphate and fluoride-based vehicles and promote subsurface remineralization, with some studies showing anticaries effects comparable to those of conventional fluoride formulations at lower fluoride levels. Because much of the benefit appears to occur within the lesion body, these systems may be especially relevant for managing initial enamel lesions (white-spot lesions). Such formulations may help limit overall fluoride exposure while preserving anticaries performance relative to conventional topical fluoride products, although longer-term clinical evidence is still emerging. A structured overview of the included *in vitro*, *in situ* and *in vivo* human studies including clinical trials is provided in Table 1.

**Table 1:** Summary of *in vitro*, *in situ* and clinical studies evaluating polyphosphate-based formulations in enamel remineralization.

Study	Study Type	Model/Sample	Intervention	Key outcomes
*In vitro*				
Lynch et al., 2006	*In vitro* (biofilm)	Bovine enamel+dentine blocks	Aqueous solution with CaGP (0.10%–0.50%)	Reduced demineralization; effect dependent on application timing
Takeshita et al., 2009	*In vitro* (pH cycling)	Bovine enamel blocks	Toothpaste with 500 ppm F + 1% TMP	Reduced mineral loss, effect similar to 1,100 ppm F at 1% TMP
Delbem et al., 2010	*In vitro* (pH cycling)	Bovine enamel blocks	Toothpaste with 1,000 ppm F + 0.25% TMP+calcium citrate	Reduced mineral loss; similar effect to 1,100 ppm F
Takeshita et al., 2011	*In vitro* (pH cycling)	Bovine enamel blocks	Aqueous solution with 3000 ppm F + 3% TMP	Reduced demineralization; lower subsurface mineral loss than fluoride alone
Danelon et al., 2012	*In vitro* (pH cycling)	Bovine enamel blocks	Gel with 4,500 ppm F + 9% SHMP	Reduced mineral loss; similar effect to higher-fluoride gels
Favretto et al., 2013	*In vitro* (pH cycling)	Bovine enamel blocks	Mouthrinse with 225 ppm F + 0.4% TMP	Reduced demineralization; improved effect at 0.4% TMP
Hirata et al., 2013	*In vitro* (pH cycling)	Bovine enamel blocks	Toothpaste with 450 ppm F + 0.25% TMP + 0.25% calcium citrate	Increased remineralization; similar effect to 1,100 ppm F
Danelon et al., 2013a	*In vitro* (pH cycling)	Bovine enamel blocks	Gel with 4,500 ppm F + 5% TMP	Reduced mineral loss; similar effect to higher-fluoride gels
Zaze et al., 2014a	*In vitro* (pH cycling)	Bovine enamel blocks	Toothpaste with 500 ppm F + 0.25% CaGP	Reduced mineral loss; similar effect to 1,100 ppm F
Da Camara et al., 2014	*In vitro* (pH cycling)	Bovine enamel blocks	Toothpaste with 250 ppm F + 0.5% SHMP	Reduced mineral loss; similar effect to 1,100 ppm F
Delbem et al., 2014	*In vitro*	Hydroxyapatite powder	Aqueous solution with 1,100 ppm F + 3% TMP	Increased CaF₂ formation and apatite precipitation
Manarelli et al., 2014	*In vitro* (pH cycling)	Bovine enamel discs	Varnish with 5% NaF + 5% TMP	Increased remineralization; greater effect vs. NaF varnish
de Castro et al., 2015	*In vitro* (pH cycling)	Bovine enamel blocks	Toothpaste with 1,100 ppm F + 3% TMP	Reduced mineral loss; greater effect vs. 1,100 ppm F
Carvalho et al., 2015	*In vitro* (pH cycling)	Bovine enamel blocks	Varnish with 2.26%–5.63% fluoride (NaF/CaF₂) + 1%–2.5% CaGP	Increased fluoride release; lower anticaries effect vs. NaF varnish
Da Camara et al., 2016	*In vitro* (pH cycling)	Bovine enamel blocks	Toothpaste with 1,100 ppm F + 1% SHMP	Reduced mineral loss; reduced subsurface lesion vs. 1,100 ppm F
Missel et al., 2016	*In vitro* (pH cycling)	Bovine enamel blocks	Toothpaste with 250 ppm F + 0.25% TMP	Reduced mineral loss; effect comparable to 1,100 ppm F
Dalpasquale et al., 2017	*In vitro* (pH cycling)	Bovine enamel blocks	Toothpaste with 1,100 ppm F + 0.5% nano-SHMP	Reduced mineral loss; increased remineralization vs. 1,100 ppm F
Danelon et al., 2017	*In vitro* (pH cycling)	Bovine enamel blocks	Toothpaste with 1,100 ppm F + 3% nano-TMP	Reduced mineral loss; increased remineralization vs. 1,100 ppm F
Gonçalves et al., 2018	*In vitro* (pH cycling)	Bovine enamel blocks	Gel with 4,500 ppm F + 9% SHMP	Increased remineralization; similar effect to higher-fluoride gels
Neves et al., 2018	*In vitro*	Bovine enamel blocks	Aqueous solution with 1% SHMP+Ca + phosphate	Increased SHMP adsorption and Ca/PO₄ uptake; promoted electron-donor sites on enamel
Mohammadipour et al., 2019	*In vitro* (modified pH cycling)	Bovine enamel	Gel with 2% NaF + 8% SHMP	Increased enamel microhardness; reduced staining
Gonçalves et al., 2020	*In vitro* (pH cycling)	Bovine enamel blocks	Toothpaste with 1,100 ppm F + 3% TMP+CPP-ACP	Reduced mineral loss; increased remineralization vs. 1,100 ppm F
Gonçalves et al., 2021	*In vitro* (pH cycling)	Bovine enamel blocks	Toothpaste with 1,100 ppm F + 3% TMP+CPP-ACP	Reduced mineral loss; increased remineralization vs. 1,100 ppm F
Nalin et al., 2021	*In vitro*	Bovine enamel blocks	Aqueous solution with 1% TMP+Ca + phosphate	Increased Ca adsorption and electron-donor sites on enamel
Sampaio et al., 2022a	*In vitro* (biofilm model)	Saliva-derived microcosm biofilms	Aqueous solution with 220 ppm F + nano-SHMP	Reduced biofilm growth and lactic acid production
Sampaio et al., 2022b	*In vitro* (biofilm model)	Dual-species biofilms (*Streptococcus mutans and Candida albicans*)	Aqueous solution with 1,100 ppm F + 1% nano-SHMP	Reduced biofilm biomass and metabolic activity; nano-SHMP showed highest antibiofilm effect
Sampaio et al., 2022c	*In vitro* (biofilm model)	Dual-species biofilms (*Streptococcus mutans and Candida albicans*)	Aqueous solution with 1,100 ppm F + 1% nano-SHMP	Altered biofilm composition; influenced Ca and P levels
Hosida et al., 2022	*In vitro* (biofilm model)	Dual-species biofilms (*Streptococcus mutans and Candida albicans*)	Aqueous solution with 1% SHMP+1,100 ppm F	Increased biofilm pH; modified inorganic composition
Amarante et al., 2023	*In vitro* (biofilm model)	Dual-species biofilms (*Streptococcus mutans and Candida albicans*)	Aqueous solution with 3% TMP+fluoride	Increased biofilm pH and extracellular matrix modification
Nagata et al., 2023	*In vitro* (pH cycling)	Bovine enamel subsurface lesions	Gel with 4,500 ppm F + 2.5% nano-TMP	Increased remineralization; reduced mineral loss vs. 4,500 ppm F; effect comparable to higher-fluoride gels
Torkasul et al., 2023	*In vitro* (pH cycling)	Human primary enamel (incisors)	Mouthrinse with 224 ppm F (SMFP) + CaGP (75 ppm Ca)	Increased microhardness and remineralization vs. fluoride alone
Gruba et al., 2024	*In vitro* (pH cycling)	Bovine enamel blocks	Toothpaste with 1,100 ppm F + TMP; plus adjunctive mouthrinse	Reduced mineral loss; increased remineralization vs. 1,100 ppm F
Oliveira et al., 2024	*In vitro* (pH cycling)	Bovine enamel blocks	Toothpaste with 1,100 ppm F + 5% TMP+CPP-ACP	Increased remineralization; reduced mineral loss vs. 1,100 ppm F
Emerenciano et al., 2024	*In vitro* (pH cycling)	Bovine enamel blocks	Toothpaste with 1,100 ppm F + 0.25% *β*-CaGP	Increased remineralization; reduced lesion depth vs. 1,100 ppm F
Nunes et al., 2024	*In vitro* (pH cycling)	Bovine enamel blocks	Toothpaste with 1,100 ppm F + 0.25% β-CaGP	Increased remineralization; reduced mineral loss vs. 1,100 ppm F
In situ				
Tenuta et al., 2009	*In situ* (crossover)	Bovine enamel blocks (in palatal appliances, human volunteers)	Toothpaste with 1,500 ppm F (SMFP) + 0.13% CaGP	No additional reduction in mineral loss vs. fluoride alone
do Amaral et al., 2013	*In situ* (crossover)	Bovine enamel blocks (palatal appliances, human volunteers)	Toothpaste with 500 ppm F + 0.25% CaGP	Reduced mineral loss; similar effect to 1,100 ppm F
Danelon et al., 2013b	*In situ* (crossover)	Bovine enamel blocks (palatal appliances, human volunteers)	Gel with 4,500 ppm F + 5% TMP	Increased remineralization; reduced mineral loss vs. lower-fluoride gels
Zaze et al., 2014b	*In situ* (crossover)	Bovine enamel blocks (palatal appliances, human volunteers)	Toothpaste with 500 ppm F + 0.25% CaGP	Increased remineralization; similar effect to 1,100 ppm F
Da Camara et al., 2015	*In situ* (crossover)	Bovine enamel blocks (palatal appliances, human volunteers)	Toothpaste with 1,100 ppm F + 1% SHMP	Reduced mineral loss; increased remineralization vs. fluoride alone
Danelon et al., 2015	*In situ* (crossover)	Bovine enamel blocks (palatal appliances, human volunteers)	Toothpaste with 1,100 ppm F + 3% nano-TMP	Increased remineralization; reduced mineral loss vs. 1,100 ppm F
Manarelli et al., 2015	*In situ* (crossover)	Bovine enamel blocks (palatal appliances, human volunteers)	Varnish with 5% NaF+5% TMP	Increased remineralization; reduced mineral loss vs. NaF varnish
Takeshita et al., 2015	*In situ* (crossover)	Bovine enamel blocks (palatal appliances, human volunteers)	Toothpaste with 500 ppm F + 1% TMP	Reduced mineral loss; similar effect to 1,100 ppm F
Nagata et al., 2016	*In situ* (crossover)	Bovine enamel blocks (palatal appliances, human volunteers)	Toothpaste with 550 ppm F + 1% TMP	No additional effect on biofilm fluoride or calcium vs. fluoride alone
Souza et al., 2016	*In situ* (crossover)	Bovine enamel blocks (palatal appliances, human volunteers)	Toothpaste with 250 ppm F + 0.05% nano-TMP	Reduced mineral loss; greater effect on subsurface lesions vs. 1,100 ppm F
Takeshita et al., 2016	*In situ* (crossover)	Bovine enamel blocks (palatal appliances, human volunteers)	Toothpaste with 500 ppm F + 1% TMP	Increased remineralization; similar effect to 1,100 ppm F
Emerenciano et al., 2018	*In situ* (double-blind crossover)	Bovine enamel blocks (palatal appliances, human volunteers)	Toothpaste with 1,100 ppm F + 3% nano-TMP	Reduced mineral loss; greater protective effect vs. 1,100 ppm F
Danelon et al., 2018	*In situ* (double-blind crossover)	Bovine enamel blocks (palatal appliances, human volunteers)	Toothpaste with 1,100 ppm F + 0.5% nano-SHMP	Increased remineralization; greater effect vs. 1,100 ppm F
Garcia et al., 2018	*In situ* (double-blind crossover)	Bovine enamel blocks (palatal appliances, human volunteers)	Toothpaste with 1,100 ppm F + 0.5% nano-SHMP	Reduced mineral loss; greater protective effect vs. 1,100 ppm F
Akabane et al., 2018	*In situ* (double-blind crossover)	Bovine enamel blocks (palatal appliances, human volunteers)	Toothpaste with 1,100 ppm F + 5% TMP	Reduced mineral loss; greater protective effect vs. 1,100 ppm F
Marcato et al., 2021	*In situ* (double-blind crossover)	Bovine enamel blocks (palatal appliances, human volunteers)	Toothpaste with 200 ppm F + 0.2% TMP+xylitol/erythritol	Reduced mineral loss; greater protection vs. 1,100 ppm F
Emerenciano et al., 2023	*In situ* (double-blind crossover)	Bovine enamel blocks (palatal appliances, human volunteers)	Toothpaste with 1,100 ppm F + 0.25% β-CaGP	Increased remineralization; greater effect vs. 1,100 ppm F
Gonçalves et al., 2024	*In situ* (double-blind crossover)	Bovine enamel blocks (palatal appliances, human volunteers)	Toothpaste with 1,100 ppm F + 10% TMP+CPP-ACP	Reduced mineral loss; greater protection vs. 1,100 ppm F
Gonçalves et al., 2025	*In situ* (double-blind crossover)	Bovine enamel blocks (palatal appliances, human volunteers)	Toothpaste with 1,100 ppm F + 10% TMP+CPP-ACP	Increased remineralization; greater effect vs. 1,100 ppm F
Martins et al., 2025	*In situ* (double-blind crossover)	Bovine enamel blocks (palatal appliances, human volunteers)	Toothpaste with 500 ppm F + 5% nano-TMP	Increased remineralization; greater effect vs. 9,000 ppm F
*In vivo* human studies				
Finn et al., 1978	*In vivo* (double-blind clinical trial, 30 months)	Children	Chewing gum with 1.5% TMP vs. gum without TMP vs. no-gum control	Reduced proximal caries increment vs. no-gum control; greater reduction with TMP nonsugar gum than TMP sucrose gum
Duke et al., 1979	*In vivo* (pilot clinical study)	Children (plaque samples)	Toothpaste with 0.8% SMFP+CaGP vs. SMFP alone	Increased plaque Ca and P vs. fluoride alone
Naylor et al., 1979	*In vivo* (double-blind clinical trial, 3-year)	Children	Toothpaste with SMFP+CaGP vs. SMFP alone vs. control toothpaste	Lower caries incidence vs. control; no significant advantage of CaGP over SMFP alone
Andlaw et al., 1983	*In vivo* (clinical trial, 3-year)	Children (11–13 years)	Toothpaste with 0.8% SMFP+3% TMP vs. SMFP alone	No reduction in caries; higher caries increment observed in TMP group vs. control
Mainwaring et al., 1983	*In vivo* (double-blind clinical trial, 4-year)	Children	Toothpaste with 1,000 ppm F (SMFP) + CaGP vs. SMFP alone	Reduced caries vs. CaGP alone; improved effect vs. SMFP alone
Sidi et al., 1989	*In vivo* (double-blind crossover)	Adults (approximal plaque samples)	Toothpaste with 1,000 ppm F (NaF, silica) vs. 1,000 ppm F (SMFP) + 0.13% CaGP (calcium carbonate) vs. fluoride-free toothpaste	Increased plaque F vs. control; CaGP^-^containing toothpaste increased Ca and inorganic phosphate vs. NaF and control
Sidi et al., 1991	*In vivo* (double-blind crossover)	Adults (approximal plaque samples)	Toothpaste with 1,000 ppm F (NaF, silica) vs. 500 ppm F (SMFP) + 1300 ppm CaGP vs. 500 ppm F (NaF) + 750 ppm CaGP vs. fluoride-free toothpaste	Increased plaque F, Ca and Pi after brushing; CaGP formulations increased Ca and Pi; effect sustained up to 24 h
Stephen et al., 1994	*In vivo* (double-blind clinical trial, 3-year)	Adolescents (11–12 years)	Toothpaste with NaF vs. SMFP vs. NaF+TMP, at 1,000 ppm F and 1,500 ppm F	No added anticaries benefit of TMP-containing toothpaste vs. fluoride controls; NaF showed slightly lower caries increment than SMFP
Städtler et al., 1996	*In vivo* (double-blind clinical trial, 3-year)	Children	Toothpaste with 3% TMP vs. toothpaste without caries-preventive agent	Reduced caries increment vs. control; greater effect with twice-daily brushing
O’Mullane et al., 1997	*In vivo* (clinical trial, 3-year)	Children	Toothpaste with 1,000 ppm F (NaF) vs. 1,500 ppm F (NaF) vs. 1,000 ppm F (NaF) + TMP vs. 1,500 ppm F (NaF) + TMP	No significant added anticaries benefit of TMP; 1,500 ppm F showed lower caries increment than 1,000 ppm F
Freire et al., 2016	*In vivo* (randomized controlled trial, double-blind)	Children	Toothpaste with 500 ppm F + 1% TMP vs. 1,100 ppm F	Reduced caries; comparable effect to 1,100 ppm F

F, fluoride; NaF, sodium fluoride; SMFP, sodium monofluorophosphate; TMP, sodium trimetaphosphate; SHMP, sodium hexametaphosphate; CaGP, calcium glycerophosphate; CPP-ACP, casein phosphopeptide–amorphous calcium phosphate; Pi, inorganic.

## 7 Limitations

Despite the promising findings supporting polyphosphate-based strategies, several limitations should be acknowledged. *In vitro* studies are often carried out on bovine enamel, which is more porous and permeable than human enamel (93–95). Moreover, pH-cycling protocols accelerate mineral exchanges and cannot replicate the complexity of oral conditions, such as biofilm maturation, salivary clearance, or dietary challenges (27, 67). *In situ* models bridge this gap but remain constrained by short intervention times and standardized sucrose exposures, which do not reflect the variability of real diets, microbiota, or lesion depths (56, 66). Even within these models, lesion characteristics strongly influence outcomes, with deeper lesions showing slower recovery (95). Clinical trials are scarce and heterogeneous, with some demonstrating caries reduction in TMP/CaGP toothpastes (19, 85), while others reported no added benefit or even adverse outcomes (90–92). Moreover, differences in formulation, fluoride:polyphosphate ratios, and application regimens across studies hinder direct comparisons and meta-analytic synthesis. Polyphosphates themselves also present intrinsic limitations. Their action is based on adsorption to enamel and modulation of ion diffusion rather than substantial direct phosphate release, making their effect most reliable when combined with external calcium and phosphate sources (73, 96). Their benefit depends on maintaining optimal fluoride:polyphosphate ratios, since excessive concentrations may impair synergy and reduce efficacy (24, 78). Phosphate salts can also reduce CaF₂ deposition on enamel, possibly due to altered fluoride precipitation dynamics in the presence of phosphate species, highlighting the need for careful formulation balance to avoid compromising superficial protection (72). CaGP^-^ containing formulations have specific challenges. When a freely available calcium source is combined with sodium fluoride (NaF), Ca^2^^+^, F^−^ and PO₄^3^^−^ may interact in the toothpaste to form insoluble compounds, potentially reducing the bioavailability of both fluoride and calcium; this concern has led most CaGP toothpastes to use sodium monofluorophosphate (SMFP) rather than NaF as the fluoride source (17). Regarding safety, TMP, SHMP and CaGP have a long history of use as food additives and cosmetic ingredients and are generally regarded as safe and well tolerated when used at the concentrations employed in oral care products (97). Available animal and human data have not demonstrated clinically relevant systemic, genotoxic or carcinogenic effects at oral or topical exposure levels comparable to those expected from topical fluoride–polyphosphate formulations, and reported adverse effects are mainly limited to local irritation at higher concentrations or in susceptible individuals (97, 98). Nano-formulations, in turn, raise additional questions about particle agglomeration, although toothpaste excipients such as glycerin and carboxymethylcellulose may stabilize dispersions (36). The long-term safety of nano-polyphosphate oral products is still uncertain, with potential risks of cytotoxicity or unforeseen side effects requiring further investigation (99).

Finally, this is a narrative review based on a focused but non-systematic literature search, without a predefined protocol, formal risk-of-bias assessment or quantitative synthesis. As a result, the possibility of selection bias cannot be excluded. The evidence base is dominated by short-term *in vitro* and *in situ* studies and a small number of heterogeneous clinical trials, which limits the strength of the inferences that can be drawn. In addition, the scope was restricted to topical fluoride–polyphosphate systems for initial enamel lesions, so extrapolation to other substrates, lesion stages or non-fluoride polyphosphate applications should be cautious.

## 8 Future perspectives

Future research should focus on combination strategies of polyphosphates with fluoride and calcium/phosphate sources, as these have shown favorable effects on remineralization and caries prevention in *in vitro* and *in situ* models. More studies are needed to determine the optimal concentrations and molar ratios for different formulations and regimens. Standardized methodologies and well-controlled clinical trials are essential to confirm consistent benefits. For nano-formulations, further investigation into long-term safety and stability is required before clinical integration (36, 99).

## 9 Conclusions

Polyphosphate-based strategies may represent a potential adjunct for enamel remineralization and caries prevention, pending confirmation from well-designed clinical trials. Available *in vitro* and *in situ* studies generally indicate synergistic interactions with fluoride and calcium/phosphate sources; however, clinical evidence remains limited. Further controlled clinical studies are required before these systems can be broadly recommended for routine use.
